# Eliciting transcriptomic and antioxidant defensive responses against Rhizoctonia root rot of sorghum using the endophyte *Aspergillus oryzae* YRA3

**DOI:** 10.1038/s41598-023-46696-7

**Published:** 2023-11-14

**Authors:** Younes M. Rashad, Mona S. Al Tami, Sara A. Abdalla

**Affiliations:** 1 Plant Protection and Biomolecular Diagnosis Department, Arid Lands Cultivation Research Institute (ALCRI), City of Scientific Research and Technological Applications (SRTA-City), New Borg El-Arab, Alexandria, 21934 Egypt; 2 Department of Biology, College of Science, Qassim University, 51452 Qassim, Saudi Arabia

**Keywords:** Plant immunity, Plant stress responses

## Abstract

Environmental pollution due to the improper use of the chemical fungicides represents a vital ecological problem, which affects human and animal health, as well as the microbial biodiversity and abundance in the soil. In this study, an endophytic fungus *Aspergillus oryzae* YRA3, isolated from the wild plant *Atractylis carduus* (Forssk.) C.Chr, was tested for its biocontrol activity against Rhizoctonia root rot of sorghum. The antagonistic potential of *A. oryzae* YRA3 was tested against *Rhizoctonia solani* in vitro. A full inhibition in the growth of *R. solani* was recorded indicating a strong antagonistic potential for this endophyte. To investigate the chemical composition of its metabolites, GC/MS analysis was used and thirty-two compounds in its culture filtrate were identified. Among these metabolites, some compounds with an antifungal background were detected including palmitic acid, 2-heptanone, and 2,3-butanediol. To these antifungal metabolites the antagonistic activity of *A. oryzae* YRA3 can be attributed. In the greenhouse experiment, treating of the infected sorghum plants with *A. oryzae* YRA3 significantly reduced severity of the Rhizoctonia root rot by 73.4%. An upregulation of the defensive genes (*JERF3*), (*POD*) and (*CHI II*) was recorded in sorghum roots when were inoculated with *A. oryzae* YRA3. In addition, an increment in the activity of peroxidase and polyphenol oxidase, as well as the total phenolic content in the sorghum roots was also recorded. Furthermore, the results obtained from the greenhouse experiment revealed a growth-promoting effect for inoculating the sorghum plants with *A. oryzae* YRA3. It can be concluded that *A. oryzae* YRA3 can be a probable biological agent to control this disease in sorghum. However, its evaluation under field conditions is highly needed in the future studies.

## Introduction

After wheat, maize, rice, and barley, sorghum (*Sorghum bicolor* (L) Moench) is the world's fifth most important cereal grain^1^. In 2021, the global cultivated area under sorghum was estimated at 40,93 million ha, which produced 61,36 million tons^2^. The archaeological evidences suggest this crop was initially grown between 5,000 and 8,000 years ago in northeastern Africa, close to the Egyptian-Sudanese border, and it spread throughout the rest of the world via trade routes^3^. Sorghum has been cultivated as a food crop for humans and as animal feed, for construction materials, or industrial raw materials. In both Africa and Asia, sorghum is an important cereal crop^4^. However, sorghum production is threatened by various pathogenic fungi that negatively affect the crop yield and growth in spite of the availability of effective chemical fungicides^5^.

*Rhizoctonia solani* Kühn represents a high global devastating pathogen, which threats many crops including sorghum and results in a high yield loss. This pathogen causes damping off, root rot, and stem canker diseases in a wide range of crops^6^. In addition, *R. solani* has the ability to persist in soil for long periods and withstands various unsuitable conditions. Various anastomosis groups (AGs) of this pathogen are known to infect different plant species, at varying degrees^7^. The disease symptoms include stunting of the plant and appearing of brown to reddish lesions along the root and stem of the plant, which may extend to girdle the stem leading to the plant death^8^. Due to the wide host range, long-term persistence in soil, and genetic variability of this pathogen, it is considered one of the dangerous plant pathogens, which results in high yield losses^9^.

In spite of the availability of some efficient chemical fungicides to control this disease^10^, these fungicides are not desired due to some health concerns. One of the most crucial global environmental problems is the incorrect use of the chemical fungicides in agriculture^11^. Heavy application of these fungicides has resulted in an environmental pollution affecting human and animal health. Moreover, the microbial equilibrium and the biodiversity of microorganisms in the soil have also been affected^12^. Therefore, researches in the recent years have focused on finding safe alternatives for the plant disease management. Biological control using beneficial microorganisms represents an eco-safe and effective solution for this problem^13,14^. Biological control of *R. solani* using different fungi and bacteria has been investigated by many researchers^15^. Rashad et al.^16^ reported an overexpression in the polyphenol biosynthesis genes in the sunflower plants that were infected by *R. solani* when were treated with the mycorrhizal fungus *Rhizophagus irregularis*.

Endophytic fungi inhabit the plant for a period of their life without causing any injury or symptoms for the plant^17^. Endophytes are known to produce unique bioactive metabolites, which enhance growth and induce tolerance of their hosts against various stresses. *Aspergillus oryzae* (Ahlb.) Cohn, a non-pathogenic and non-aflatoxigenic fungus that has been widely used in many fermentation industries such as enzymes production for baking. Furthermore, this fungus and its enzymes are accepted by FAO/WHO as food additives (probiotics), as well as in poultry^18^. Due to its high secreting ability, *A. oryzae* has been reported as an efficient cell factory for organic acids, industrial enzymes, and secondary metabolites along the past few decades^19^. It has various roles in the fields of food, therapeutics, forage, and the ecosystems^20^. Few researches have investigated the biocontrol activity of *A. oryzae* against various plant pathogens. In this concern, Alshannaq et al.^21^ reported an inhibitory effect for *A. oryzae* M2040 against production of aflatoxin B1 and growth of the toxigenic fungus *A. flavus* in vitro and on peanuts. The study reported also the production of *A. oryzae* M2040 for some antifungal compounds as a suggested mode of action. Furthermore, the nematicidal activity against *Meloidogyne incognita* was also reported for *A. oryzae*^22^.

This work was aimed at (1) studying the antifungal activity of the endophyte *A. oryzae* against *R. solani*, (2) detecting its antifungal metabolites, and (3) evaluating its biocontrol activity against *Rhizoctonia* root rot of sorghum.

## Results

### Molecular identification of the endophytic fungus YRA3

The endophytic fungus YRA3 was molecularly identified using the ITS region of the DNA. Data obtained from the NCBI BLAST based on the ITS sequence revealed that the endophytic fungus YRA3 had 100% similarity with that of *A. oryzae* (MK714921) confirming the identity of the endophytic fungus YRA3 as *A. oryzae* YRA3. The nucleotide sequence of *A. oryzae* YRA3 (584 bp) was deposited in the GenBank under the accession No. (OQ916424). The phylogenetic relationship with the closest species in the genus *Aspergillus* is illustrated in Fig. 1. The phylogenetic tree showed that *A. oryzae* YRA3 was clustered with *A. oryzae* (MK714921) in a distinct subcluster with 89% bootstrap support. This subcluster was grouped with 89% bootstrap support with *A. flavus* (HQ285616). In the same time, *A. niger* (HM136829) and *A. fumigatus* (EU664466) were separately grouped in distinct subclusters, while *A. sydowii* (NR_131259) and *A. nidulans* (AY373888) were grouped in one subcluster. *Aspergillus terreus* (OP113794) was clustered as an outgroup taxon.

**Figure ﻿1:**
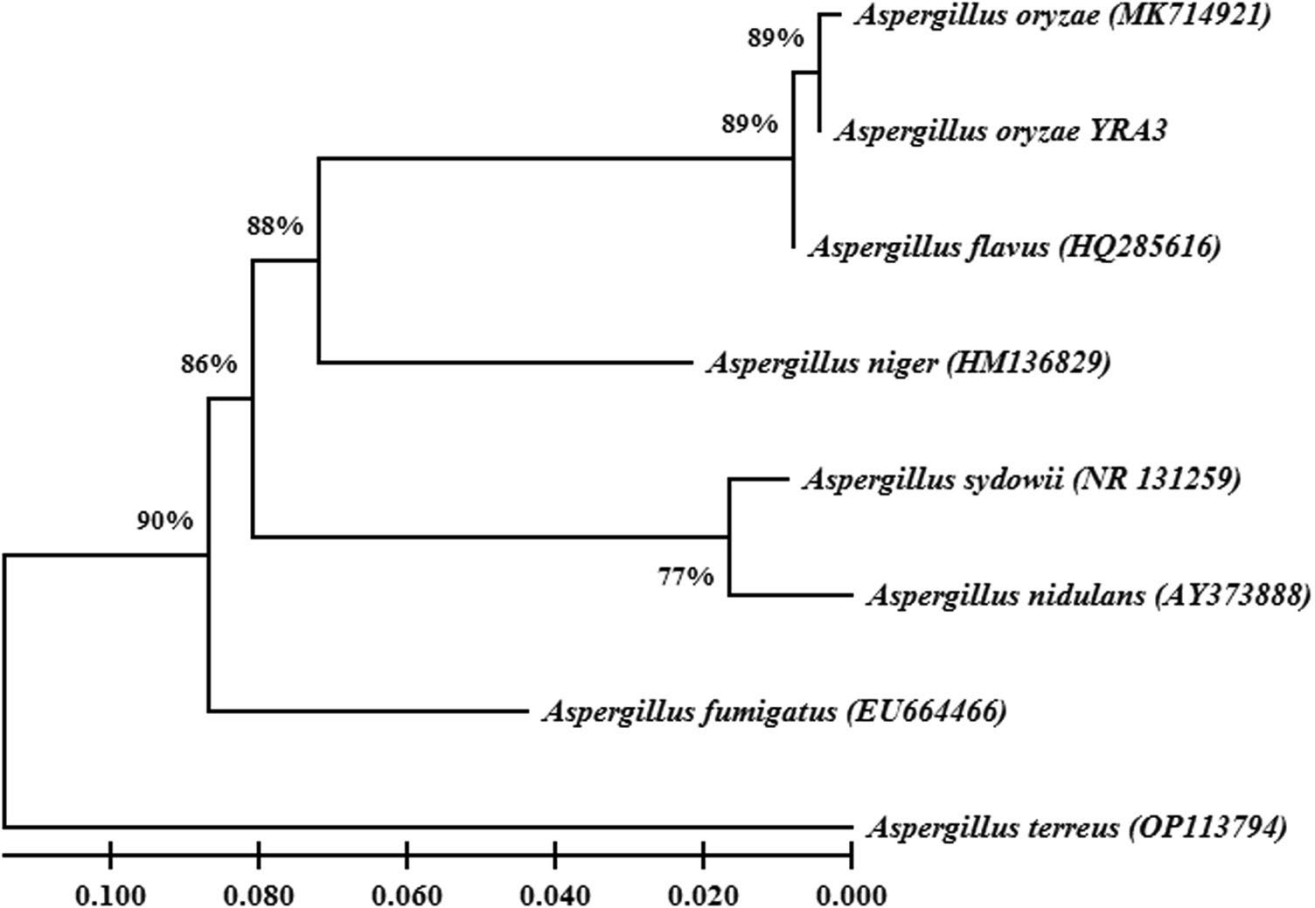
Phylogeny tree indicates the relationship between *Aspergillus oryzae* YRA3 and the closest species in the genus *Aspergillus*. At each node, the bootstrap value (%) is shown. The scale bar reveals number of the nucleotide substitutions per site.

### Antifungal assay of *A. oryzae* YRA3 against *R. solani*

**Figure 2:**
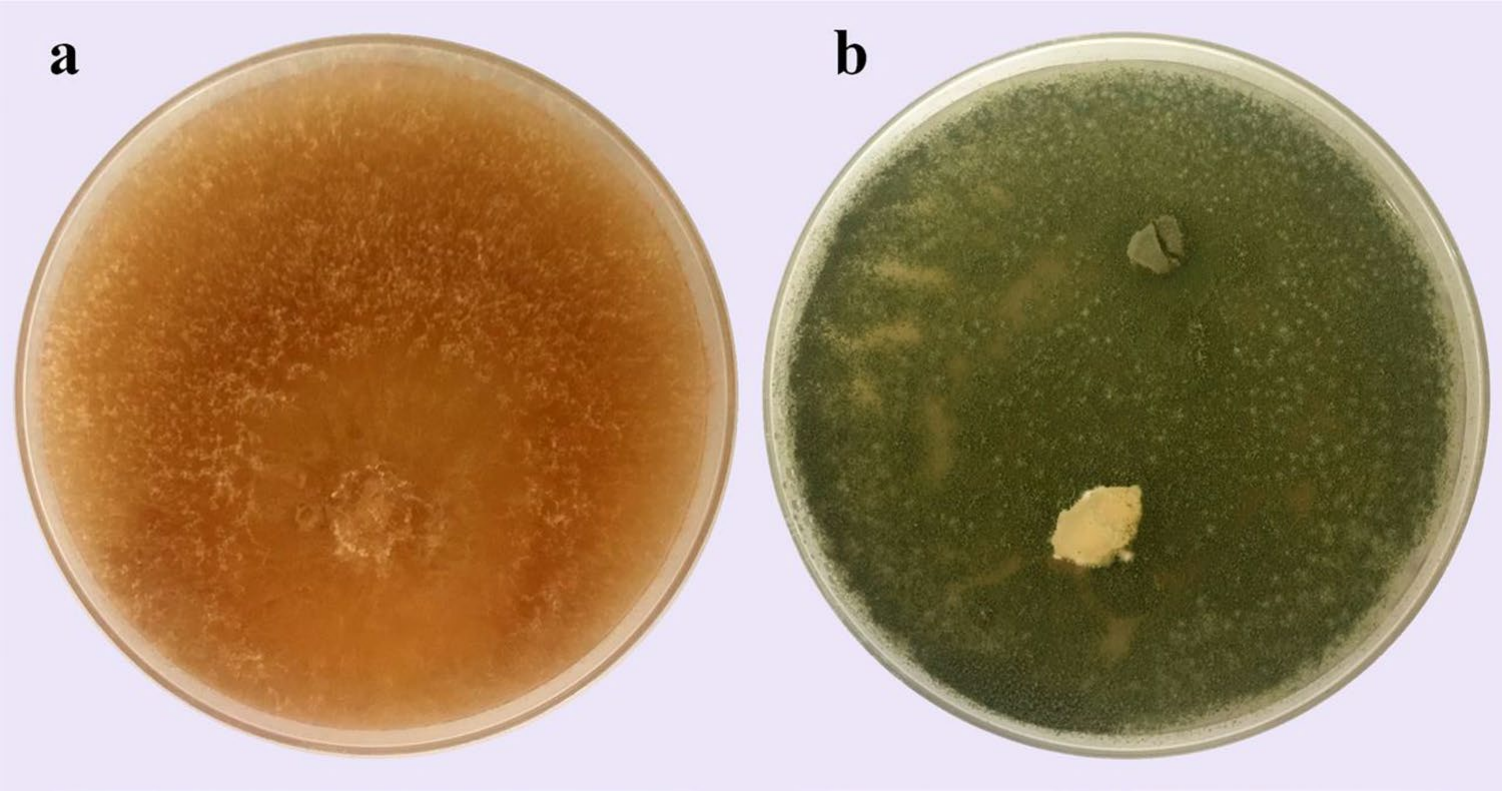
Dual culture test shows the antagonistic behavior of *Aspergillus oryzae* YRA3 against *Rhizoctonia solani,* where **(a**) control plate of *R. solani,* and **(b**) the dual culture plate**.**

The antagonistic potential of *A. oryzae* YRA3 was assessed against *R. solani* in vitro (Fig. 2). A fast growth rate for *A. oryzae* YRA3 was observed, compared to *R. solani* in the second day of incubation*.* Three days of incubation, a full inhibition in the mycelial growth of *R. solani* and a full growth of *A. oryzae* YRA3 were recorded in the dual culture plate, compared with the control. The obtained result indicated a strong antagonistic activity of *A. oryzae* YRA3 against *R. solani*.

### Gas chromatography/mass spectrometry (GC/MS)

**Figure 3:**
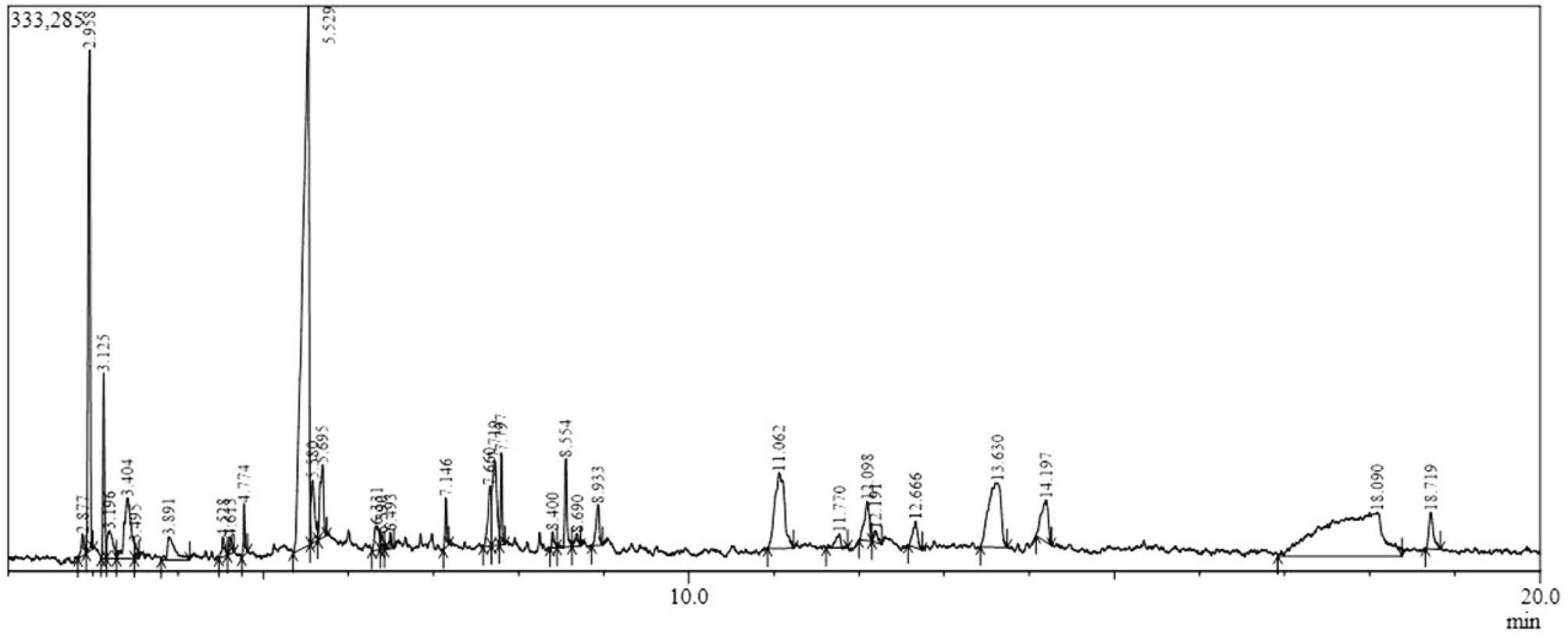
A GC/MS chromatogram illustrates the chemical composition of the cultural filtrate of *A. oryzae* YRA3.

The culture filtrate of *A. oryzae* YRA3 was analyzed by a GC/MS system to identify its chemical composition. Thirty-two substances were detected at different percentages (Fig. 3 and Table 1). The major contents included palmitic acid (25.1%), 2-heptanone (19.35%), pyrrolo[1,2-a]pyrazine-1,4-dione, hexahydro (17.4%), and 2,3-butanediol (14.3%). In addition, other constituents were detected at intermediate percentages such as 3-methyl-5-propylnonane (3.3%), heneicosane (2.81%), glyceraldehyde (2.6%), and 3-deoxy-d-mannoic lactone (1.9%). While the other constituents were found at minor extents.

**Table 1:** Chemical composition of the culture of *A. oryzae* YRA3.

Peak #	Compound name	Peak area (%)	Retention time (min)
1	n-Octadecane	0.28	2.877
2	1-Hexadecanol	1.4	2.958
3	Heneicosane	2.81	3.125
4	1,2-Propanediol	0.8	3.196
5	2,5-Piperazinedione, 3,6-bis(2-methylpropyl)	1.04	3.404
6	3 Isopropoxy 1,1,1,7,7,7 hexamethyl 3,5,5	0.36	3.495
7	Heptasiloxane, hexadecamethyl-	0.44	3.891
8	Ethanol, 2-(2-aminoethoxy)-	0.19	4.528
9	1-Chlorooctadecane	0.31	4.615
10	7,9 Ditertbutyl-1-oxaspiro (4,5) deca 6,9	0.89	4.774
11	Pyrrolo[1,2-a]pyrazine-1,4-dione, hexahydro	17.4	5.529
12	Palmitic acid	0.29	5.380
13	Methyl 3 (3,5 ditertbutyl 4 hydroxyphenyl) propionate	0.22	5.695
14	3-Methyl-5-propylnonane	3.3	6.331
15	Propanamide	0.35	6.493
16	3-Cyano-3-octyl-1,4-cyclohexadiene	0.45	7.146
17	Eicosane	0.27	7.660
18	2-Hexyldecanol	0.68	7.718
19	Glyceraldehyde	2.6	7.797
20	Malonic acid diamide	0.12	8.400
21	8,14-Cedranoxide	0.37	8.554
22	3,5-di-tert-Butyl-ortho-benzoquinone	0.11	8.690
23	α-Linolenic acid	0.15	8.933
24	2,3-Butanediol	14.3	11.062
25	Octadecanoic acid	1.04	11.770
26	1,3-Dioxolan-2-yl-N-methylmethanamine	0.71	12.098
27	2,4-Di-tert-butylphenol	0.95	12.191
28	Tetrakis(trimethylsiloxy)silane	1.01	12.666
29	2-heptanone	19.35	13.630
30	3-Deoxy-d-mannoic lactone	1.9	14.197
31	Palmitic acid	25.1	18.090
32	L-Proline, N-valeryl-, octadecyl ester	0.81	18.719

### Effect of inoculating the sorghum plants with *A. oryzae* YRA3 on the disease severity

Biocontrol activity of *A. oryzae* YRA3 against Rhizoctonia root rot of sorghum was investigated under the greenhouse conditions. The obtained results showed that sorghum plants, which not received *R. solani* inoculum, did not show any disease symptoms. On the contrary, sorghum plants that were only inoculated with *R. solani* showed 73% severity. In the same time, the infected sorghum plants, which were inoculated with *A. oryzae* YRA3 showed 19.4% reduction in the disease severity than the infected plants, which were not inoculated with *A. oryzae* YRA3.

### Effect of inoculating the sorghum plants with *A. oryzae* YRA3 on the transcriptional expression of the defensive genes

**Figure 4:**
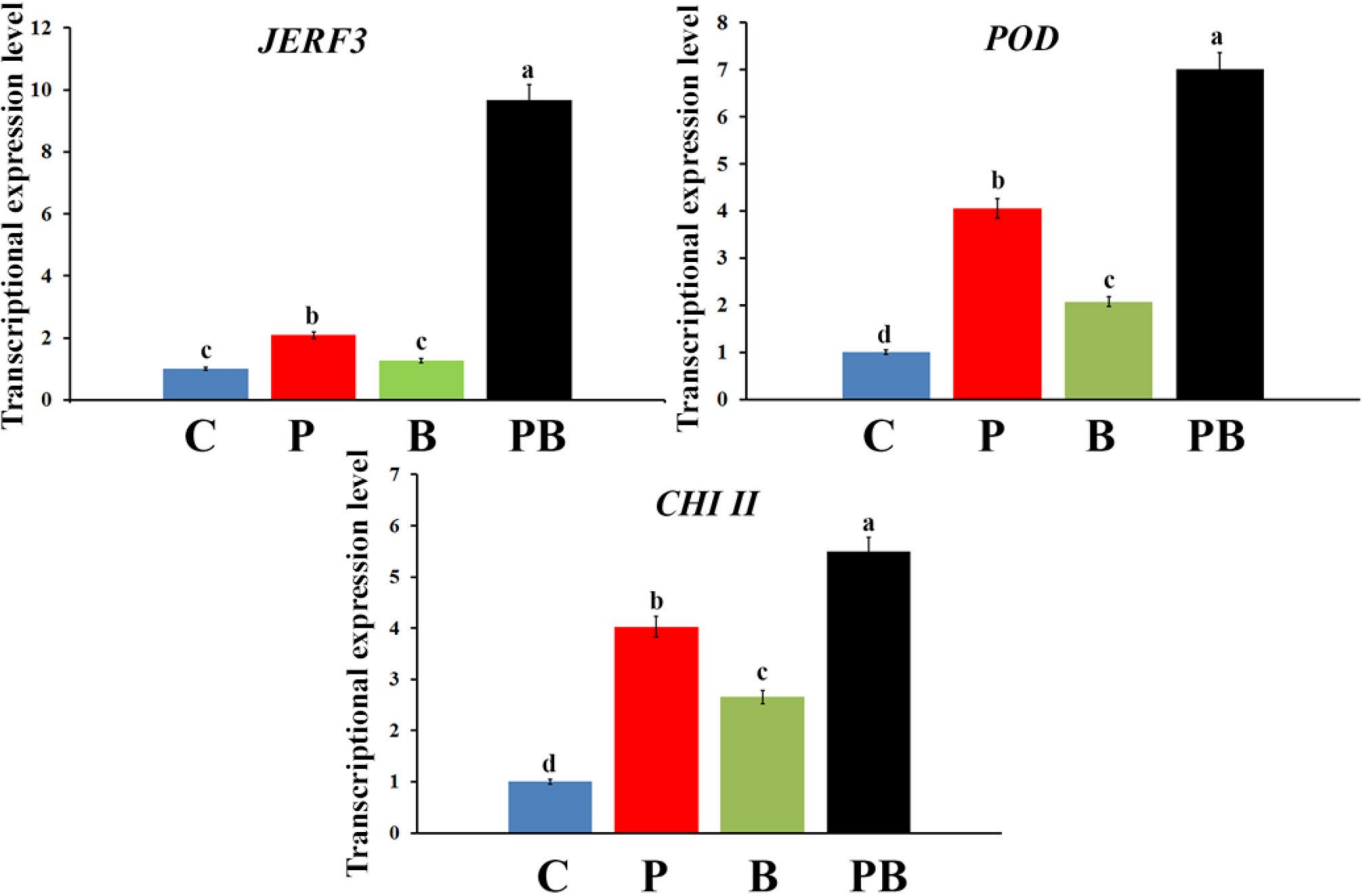
The expression level of jasmonate and ethylene-responsive factor 3 (*JERF3*), peroxidase (*POD*), and chitinase II (*CHI II*) genes in sorghum roots that were infected with Rhizoctonia root rot and/or inoculated with *A. oryzae* YRA3. Where, C = non-inoculated and non-infected sorghum plants (control), P: infected and non-inoculated with *A. oryzae* YRA3, B: uninfected and inoculated with *A. oryzae* YRA3, and PB: infected and inoculated with *A. oryzae* YRA3. In each subfigure, based on Tukey’s HSD test at *p* ≤ 0.05, no significant difference is recorded between columns that are subscripted with the same letter. Standard error of each value is illustrated by the error bar.

The transcriptional expression of jasmonate and ethylene-responsive factor 3 (*JERF3*), peroxidase (*POD*), and chitinase II (*CHI II*) in sorghum roots, which were infected with *R. solani* and/or treated with *A. oryzae* YRA3 is presented in Fig. 4. The obtained data showed that inoculation of the sorghum plants with *R. solani* (P) overexpressed *JERF3,* while no significant change in its expression level was observed in response to their inoculation with *A. oryzae* YRA3 (B), compared to the control plants (C). The highest expression level for this gene was recorded for sorghum plants, which were inoculated with *R. solani* and *A. oryzae* YRA3, recording 9.7-fold. The results indicated that inoculation of sorghum plants with *R. solani* and/or *A. oryzae* YRA3 upregulated *POD*, at varying extents, compared with the control plants. However, *R. solani* had more inducing effect than *A. oryzae* YRA3 in this concern. The highest expression level of *POD* was recorded for the sorghum plants, which were inoculated with *R. solani* and *A. oryzae* YRA3, recording 7-fold. For *CHI II*, the obtained results showed that inoculation of the sorghum plants with *R. solani* and/or *A. oryzae* YRA3 considerably triggered *CHI II*, at varying degrees, compared to the negative control treatment. However, *R. solani* was more effective than *A. oryzae* YRA3 in this regard. The highest expression level of *CHI II* was recorded for the sorghum plants, which were inoculated with *R. solani* and *A. oryzae* YRA3, recording 5.5-fold.

### Effect of inoculating the sorghum plants with *A. oryzae* YRA3 on the plant growth

Table 2 presents the growth parameters of the sorghum plants, which were inoculated with *R. solani* and/or *A. oryzae* YRA3. Compared to the control plants, results from the greenhouse experiment indicated that inoculation with *R. solani* led to a significant reduction in most of the evaluated growth parameters (shoot and root lengths and shoot and root dry weights), but not on number of leaves. On the contrary, inoculating the sorghum plants with *A. oryzae* YRA3 significantly promoted most of the evaluated growth parameters, compared to the control, recording the highest values in this regard. In addition, inoculating of the infected sorghum plants with *A. oryzae* YRA3 significantly enhanced their growth parameters, except for the number of leaves, compared with that of the infected plants, which were not inoculated with *A. oryzae* YRA3.

**Table 2:** Mean growth parameters of sorghum plants, which were infected with *R. solani* and treated with *A. oryzae* YRA3*.

Treatment	Shoots length (cm)		Root length (cm)	Shoots dry weight (g)	Root dry weight (g)	Number of leaves
C	54.2 ± 1.2^b^	17.1 ± 0.5^b^		1.0 ± 0.04^ab^	0.55 ± 0.04^b^	5.0 ± 0.1^a^
P	39.0 ± 1.4^c^	12.5 ± 0.4^c^		0.83 ± 0.02^c^	0.43 ± 0.06^c^	5.0 ± 0.1^a^
B	63.4 ± 1.1^a^	18.9 ± 0.3^a^		1.1 ± 0.04^a^	0.62 ± 0.02^a^	6.0 ± 0.2^a^
PB	51.8 ± 1.3^b^	16.7 ± 0.8^b^		0.91 ± 0.03^b^	0.51 ± 0.03^b^	5.0 ± 0.2^a^

*Values that are followed by the same letter in each column are not significantly different according to Tukey's HSD test (*p* ≤ 0.05), values are the means of 4 replicates ± SD.

### Effect of inoculating the sorghum plants with *A. oryzae* YRA3 on the total phenols and activity of the antioxidant enzymes

Total phenols and activity of the antioxidant enzymes peroxidase (POD) and polyphenol oxidase (PPO) in the infected sorghum plants that were inoculated with *A. oryzae* YRA3 are presented in Table 3. Data revealed that inoculation of the sorghum plants with *R. solani* led to an increment in the total phenols content as well as the activity of POD and PPO. In the same time, inoculating of the sorghum plants with *A. oryzae* YRA3 led to an increment in these parameters. However, the priming effect due to inoculating with *R. solani* was higher than that due to *A. oryzae* YRA3. The highest values in these parametrs were recorded for sorghum plants, which were inoculated with *R. solani* and *A. oryzae* YRA3, recording 312.8 ± 3.1 mg.g^-1^ fresh weight for the total phenols content and 3.59 ± 0.2 ∆A_470_ min^−1^ g^−1^ fresh weight and 2.69 ± 0.1 ∆A_420_ min^−1^ g^−1^ fresh weight for activity of POD and PPO, respectively.

**Table 3:** Mean total phenols and activity of peroxidase and polyphenol oxidase of sorghum plants infected with *R. solani* in response to treating with *A. oryzae* YRA3*.

Treatment	Total phenols (mg.g^−1^ fresh wt)	Peroxidase (∆A_470_ min^−1^ g^−1^ fresh wt)	Polyphenol oxidase (∆A_420_ min^−1^ g^−1^ fresh wt)
C	120.3 ± 2.4^d^	1.15 ± 0.1^d^	1.43 ± 0.3^d^
P	283.4 ± 3.3^b^	3.12 ± 0.2^b^	2.41 ± 0.2^b^
B	220.6 ± 2.8^c^	2.63 ± 0.3^c^	1.98 ± 0.2^c^
PB	312.8 ± 3.1^a^	3.59 ± 0.2^a^	2.69 ± 0.1^a^

*In each column, values followed by the same letter are not significantly different according to Tukey's HSD test (*p* ≤ 0.05), each value represents the mean of four replicates ± SD.

## Discussion

Rhizoctonia root rot is a destructive worldwide disease, which affects a wide host range resulting in a high yield loss^6,7^. Researches in the recent years have indicated that many of the endophytic fungi and bacteria have been recognized as promising biocontrol agents against various phytopathogenic fungi^23–25^. In this study, the antagonistic activity of *A. oryzae* YRA3 was assessed against the mycelial growth of *R. solani* in vitro. The obtained results showed a very strong antagonistic activity resulting in a complete inhibition of *R. solani* growth*.* This result is in agreement with that of Houda Sara et al.^26^ who reported an antifungal activity of the culture extract of *A. oryzae* against *Candida albicans* as well as an antibacterial activity against a wide range of bacteria*.* This result can be explained in the light of two modes of action: the first is the competition of *A. oryzae* YRA3 for the space and/or nutrients. The fast growth rate of *A. oryzae* YRA3 compared to that of *R. solani*, which was recorded in this study supports this mechanism. The second probable mechanism is the antibiosis via production of hydrolytic enzymes and/or antifungal metabolites. Endophytic fungi are considered an important source of unique bioactive metabolites^27^. Around 20,000 natural and bioactive products have been described from different endophytic microorganisms^28^. This ability can be attributed to the variation in the ecological niches in which these endophytes inhabit and the biotic and/or abiotic stresses they face^29^. To prove this hypothesis, a GC/MS analysis of the culture filtrate of *A. oryzae* YRA3 was carried out. Among the detected 32 compounds, different bioactive metabolites with antifungal background were identified, to them the antifungal behavior of *A. oryzae* YRA3 can be attributed. Among them, 2-heptanone, palmitic acid, heneicosane, and 2,3-butanediol are known to have an antifungal activity against different fungi^30,31^. In this concern, Wu et al.^32^ reported the antifungal activity of 2-heptanone against *Fusarium oxysporum*, the causal of Fusarium wilt of watermelon. Different antifungal mechanisms have been discussed in this regard such as increasing electrolyte leakage of the pathogen cell via disturbance of the cell membrane permeability and suppression of protein synthesis^33^.

Under the greenhouse conditions, a significant suppression in the Rhizoctonia root rot severity was achieved in the infected sorghum plants that were inoculated with *A. oryzae* YRA3, indicating the probable biocontrol activity of this endophyte. To investigate effect of application of this endophyte on the plant immunity, the transcriptional expression of three defense-related genes (*JERF3*, *POD*, and *CHI II*), that represent different signaling pathways, was studied. qPCR results indicated that *JERF3*, *POD*, and *CHI II* were significantly upregulated due to inoculation with *A. oryzae* YRA3. This indicates the resistance-inducing effect of *A. oryzae* YRA3 on the sorghum plants, particularly in case of the infection with *R. solani*. *JERF3* is a responsive factor, which regulates a set of the resistance genes through both jasmonic acid and ethylene signaling pathways resulting in an induction of the plant immunity against the fungal infection^34^. This means that the recorded overexpression of *JERF3* in this study may led to triggering of other defensive genes in the inoculated sorghum plants. This information was supported by the recorded upregulation of *POD*, and *CHI II* in this study that was associated with the *JERF3* upregulation. *CHI II* gene encodes for the antifungal chitinase enzyme. This enzyme plays a crucial role in degrading the chitin, the main structural unit in the fungal cell wall^35^. Degradation of the chitin units in the fungal cell wall leads to a disturbance in the cell permeability, leakage of the cytoplasmic electrolytes, and finally the cell death^36^. The recorded upregulation of *CHI II* in this study due to application of *A. oryzae* YRA3 revealed the inducing effect of this endophyte on the plant resistance against the penetration of *R. solani* to the sorghum roots*.* The antioxidant gene *POD* encodes the peroxidase enzyme, which has an antioxidant role against the free radicals and the reactive oxygen species (ROS) resulted due to the different biotic or a biotic stresses^37^. Upregulation of *POD* was supported also by the recorded increment in the levels of the activity of the antioxidant enzymes (POD and PPO) in the infected sorghum plants that were inoculated with *A. oryzae* YRA3, compared with the untreated-infected plants. These antioxidant enzymes act as scavengers for the destructive free radicals and ROS that severely destroy the infected plant cells^38^. The observed overexpression of *POD* due to application of *A. oryzae* YRA3 indicated the inducing effect of this endophyte on the resistance of sorghum plants against fungal infection. The recorded overexpression of these defense-related genes indicated another indirect biocontrol mechanism of the endophyte *A. oryzae* YRA3.

One of the most interesting results obtained in this study is the increment in the content of the phenolic compounds in the sorghum roots due to the inoculation with *A. oryzae* YRA3. The phenolic compounds are very fungi-toxic substances that naturally produced in the plants as a defense line against the fungal penetration and to prevent the transfer of the pathogen from cell to cell^39^. The increment in the phenolic content is usually used as a marker for the induction of the plant resistance^40^. In the line with this result, El-Sharkawy et al.^41^ reported an increment in the phenolic compounds in the wheat plants when were inoculated with the endophyte *Epicoccum nigrum* HE20 against stripe rust. Different mechanisms are known to be involved in priming plant resistance via endophytes such as thickening of the cell walls, phytoalexins secretion and synthesis of PR-proteins, which play a restricting role against the pathogen penetration and development^42^. Furthermore, a previous study reported that 2,3-butanediol, which detected in this study as a metabolite of *A. oryzae* YRA3, can induce the production of the root exudates which modulate the growth of the rhizospheric fungi and bacteria^43^. In addition, our results showed a growth-promoting effect for the application of *A. oryzae* YRA3 on the sorghum plants. The results showed a production of 2,3-butanediol in the culture filtrate of *A. oryzae* YRA3 that is known as a growth enhancer in many plant species^43^. This result is in agreement with that reported by Wu et al.^32^ who reported the growth-promoting effect of 2,3-butanediol on *Arabidopsis thaliana* plantlets. Production of this substance may explain the observed growth-promoting effect of *A. oryzae* YRA3.

## Conclusion

These results demonstrated that *A. oryzae* YRA3, isolated from the wild plant *Atractylis carduus,* has an antagonistic potential against *R. solani* in vitro. In addition, this endophyte showed a biocontrol activity under the greenhouse conditions, which may qualify *A. oryzae* YRA3 as a promising candidate to control Rhizoctonia root rot of sorghum as well as a plant growth-promotor. However, further investigations are still needed to evaluate its efficacy under the field conditions.

## Materials and methods

### Sorghum cultivar and used fungi

Sorghum grains (cv. Shandaweel-1), obtained from Agricultural Research Center, Egypt, were used during the greenhouse evaluation. A virulent strain of *R. solani* (AG-2–2 IIIB) was kindly obtained from Agricultural Research Center, Egypt. The fungus was maintained on potato dextrose agar medium (PDA) at 4 °C. The fungal inoculum was prepared by culturing the phytopathogenic fungus *R. solani* on a sterilized maize/soil medium (1:3, v/v) for 15 days at 30 °C.

### Isolation and identification of the endophytic fungus

The endophytic fungus was isolated from the wild plant *Atractylis carduus* (Forssk.) C.Chr., which was collected from a semi desert area in Borg EL-Arab, Alexandria, Egypt. Different pieces of the wild plant (stem, roots, and leaves) were surface sterilized by immersing in NaOCl (0.5%) for 2 min, followed by immersing in ethanol (70%) for 2 min, then washing in dist. water. The surface sterilized plant pieces were put on PDA plates (4 pieces per plate) and incubated at 30 °C for 72 h. The recovered fungus was purified using the hyphal tip technique and morphologically identified based on its microscopic and cultural properties according to Pitt et al.^44^. The wild plant was identified by Dr. Ahmed Abd El-gawad, and deposited at the Herbarium of Faculty of Agriculture, Mansoura University under the deposition number (AW 1318/22/102).

### Molecular identification

The endophytic fungus YRA3 was molecularly identified by amplifying the internal transcribed spacer (ITS) part in the DNA. Extraction of the total DNA was performed using QiAamp DNA Kit (Qiagen, Germany). The universal primers ITS1 (5’TCC GTA GGT GAA CCT TGC GG3’) and ITS4 (5’TCC TCC GCT TAT TGA TAT GC3’) were used to amplify the ITS region (600 bp) according to Madbouly et al.^23^. To identify the endophytic fungus YRA3, the produced sequence was compared with various sequences in NCBI database. Sequence of the endophyte YRA3 and the closest ones from the GenBank database were aligned using the ClustalW algorithm and the phylogeny tree was constructed using the neighbor-joining method via MEGAX-10.2.4^45^.

### Assay of the antagonistic potential of *A. oryzae *YRA3 against *R. solani* in vitro

The antagonistic potential of *A. oryzae* YRA3 was tested against *R. solani* in vitro using the dual culture method. On a PDA plate, a 7 mm-diameter disc of *A. oryzae* YRA3, taken from a 5-days-old culture, and another disc from a 5-days-old culture of the pathogen were inoculated (7 cm) in opposite to each other in the plate. Plates singly inoculated with each fungus served as a control treatment. The PDA plates were incubated at 28 °C for a week. The mycelial growth of the growing fungi and the inhibition percentage were measured.

### GC/MS analysis

The chemical composition of the cultural filtrate of *A. oryzae* YRA3 was analyzed using a GC/MS-QP2010 system (Shimadzu, Japan). The DB-5HT capillary column (30 m, 0.32 mm, and 0.10 µm,) was used. The programed oven temperature was as follows: 50 °C/1 min, 180 °C/1 min, 230 °C/2 min, and then 250 °C. All compounds were identified according to the NIST 11 Spectral Library (Gaithersburg, MD, USA).

### The greenhouse experiment

Surface sterilized sorghum grains (using hypochlorite solution 5% and ethanol 70%, 1 min for each) were planted in plastic pots filled with sterilized sandy-clay soil (sand:clay 2:1 *v/v*) at 5 grains per pot. To prepare the inoculum of *A. oryzae* YRA3, the fungus was grown on PDA at 28 °C for a week. A conidial suspension was prepared in sterile water, mixed and adjusted at 10^6^ conidia mL^−1^. Sorghum grains soaked in the conidial suspension of *A. oryzae* YRA3 for 2 h were planted in plastic pots at 5 grains per pot. Soil infestation was done 14 days post planting by mixing the pathogen inoculum with the upper layer of the soil at 2.5%. Four replicates of each treatment were applied. The pots were irrigated when necessary, and arranged in a complete randomized design in a greenhouse (31/20 °C day/night and 73% humidity). The treatments in this experiment were as follows: C = untreated control, P: infected with *R. solani*, B: uninfected-treated with *A. oryzae* YRA3, and PB: infected and treated with *A. oryzae* YRA3.

### Quantification of the transcriptional expression level of the defense-related genes

Seven days after infection, sorghum roots of each treatment were sampled. Extraction of the total RNA was done using RNeasy Kit (Qiagen, Hilden, Germany). cDNA of the extracted RNA was synthesized using a SureCycler 8800 thermocycler (Agilent, Santa Clara, CA, USA). The reaction composed of 20 μL containing total RNA (3 μL), buffer (2.5 μL), primer (5 μL), reverse transcriptase (0.2 μL), dNTPs (2.5 μL), and RNase-free water (6.8 μL). The program was performed at 42 °C/1 h, and at 70 °C/10 min. The real-time PCR was done by a Rotor Gene system (Qiagene, USA). The reaction 20 μL composed of 3 μL cDNA, 12.5 μL SYBR Green Master Mix (Bioline, Germany), 1.5 μL of each primer, and 1.5 μL RNase-free water. The used primers are presented in Table 4. The reference gene utilized was *β*-actine. The qPCR program was as follows: 1 cycle (95 °C/3 min), 45 cycles (95 °C/15 s, 56 °C/30 s, and 72 °C/30 s).

**Table 4:** The used primer sequences.

Gene description	Abbrev	Sequence (5′-3′)
Jasmonate and ethylene-responsive factor 3	*JERF3*-F *JERF3*-R	GCCATTTGCCTTCTCTGCTTC GCAGCAGCATCCTTGTCTGA
Peroxidase	*POD*-F *POD*-R	CCTTGTTGGTGGGCACACAA GGCCACCAGTGGAGTTGAAA
Chitinase class II	*CHI ΙΙ*-F *CHI ΙΙ*-R	GCGTTGTGGTTCTGGATGACA CAGCGGCAGAATCAGCAACA
*β*-actin (reference gene)	*β*-actin-F *β*-actin-R	GTGGGCCGCTCTAGGCACCAA CTCTTTGATGTCACGCACGATTTC

### Disease evaluation

To evaluate the disease severity, five sorghum roots of each treatment were collected 45 days post infection and rated according to a 5-degrees scale of Carling et al.^46^, where 0: no disease, 1: discoloration without necrosis, 2: discoloration with low necrosis, 3: discoloration with high necrosis, and 4: complete death. The following equation was used to calculate the disease severity^47^:

$${\text{DS }} = { }\left( {1{\text{n}}1{ } + { }2{\text{n}}2{ } + { }3{\text{n}}3{ } + { }4{\text{n}}4{ } + { }5{\text{n}}1/{ }5{\text{N}}} \right){ } \times { }100$$

where; n_1_: the number of plants with score 1; n_2_: the number of plants with score 2; etc.; n_5_: the number of plants with score 5; and N: total number of plants used in the experiment.

### Plant growth evaluation

For each treatment, five sorghum plants were uprooted, washed under tap water, and evaluated for the growth parameters (lengths and dry weights of plant shoot and root, and leaves number). The plant samples were dried at 85 °C for 72 h before be weighted.

### Total phenols and enzymes activity

Total phenols and activity of POD and PPO enzymes were estimated 45 days post infection in the sorghum roots. For extraction, 0.5 g of the sorghum root was homogenized in 5 mL ethanol (80%), then centrifuged at 3000 rpm for 20 min. The supernatant was collected and evaporated to the dryness. The residue was dissolved in 5 mL distilled water and used for estimation of the total phenols according to Malik and Singh^48^. Activity of POD was determined according to Maxwell and Bateman^49^, while activity of PPO was determined as described by Galeazzi et al.^50^.

### Statistical analyses

The data were statistically analyzed using the CoStat software^51^. Comparing of the means were done based on Tukey’s HSD test at *p* ≤ 0.05.

### Ethical approval

Authors confirm that all the methods and experiments in this study, including the collection of plant material, complied with the institutional, national, and international guidelines and legislation.

## Data Availability

Nucleotide sequence of *A. oryzae* YRA3 (584 bp) was deposited in the NCBI database under the accession No. (OQ916424) https://www.ncbi.nlm.nih.gov/nuccore/OQ916424.
